# Editorial: Economic and health impacts of dietary interventions

**DOI:** 10.3389/fnut.2023.1283108

**Published:** 2023-09-19

**Authors:** Eliseu Verly-Jr, Isis Eloah Machado, Eduardo Augusto Fernandes Nilson

**Affiliations:** ^1^Department of Epidemiology, Rio de Janeiro State University, Rio de Janeiro, Brazil; ^2^Medicine School, Federal University of Ouro Preto, Ouro Preto, Brazil; ^3^Núcleo de Pesquisas Epidemiológicas em Nutrição e Saúde, São Paulo, Brazil

**Keywords:** diet cost, Burden of Diseases, dietary interventions, cost-effectiveness, health impacts

Much progress has been achieved in the understanding of the effect of food and nutrients on preventing non-communicable chronic diseases (1). Dietary interventions and policies aim at improving diet habits in the population taking into account evidence-based relationships between dietary risk factors and disease burden. However, the implementation of the interventions and policies, in general, demands investments from the individuals or the government, and social and cultural characteristics and income are essential barriers that should be accounted for when planning populational dietary interventions (2). On the other hand, the potential benefits, such as preventing premature deaths and saving health care costs may overcome the investments. The economic, epidemiologic, and social impact are then necessary dimensions in policy and intervention evaluation, for which theoretical and methodological challenges have been an object of study by several research groups. Some of these aspects were addressed in this Research Topic with high-quality and relevant papers as summarized below.

The papers, titled “*Estimating the dietary impact and health benefits of front-of-pack labeling regulations in Canadian adults*” and “*The estimated dietary and health impact of implementing the recently approved ‘high in' front-of-package nutrition symbol in Canada: a food substitution scenario modeling study*” examines the potential impact of recently introduced dietary regulations on promoting healthy diets and reducing deaths in the Canadian population (Flexner, Ng, et al.; Flexner, Ahmed, et al.). Using different contrafactual scenarios, both studies show that Front-of-Pack Labeling (FOLP), by significantly reducing sodium, total sugar, and saturated fat intakes among Canadians, has the potential to prevent or postpone ~70% of diet-related NCD deaths in the country. This studies' findings provide critical evidence for policymakers and health authorities from all over the world considering the potential implementation of FOPL regulations.

Two other papers from this supplement, entitled “*Health economic impacts associated with the consumption of sugar-sweetened beverages in Brazil*” and “*Chronic diseases attributable to a diet rich in processed meat in Brazil: burden and financial impact on the healthcare system*” were based on ecological studies using data the Global Burden of Disease (GBD) to estimate the direct economic costs and the burden of non-communicable diseases associated with selected dietary risk factors in Brazil (Leal et al.; Rocha et al.). Both studies demonstrated the relevant health and epidemiological impact of these dietary risk factors among Brazilian adults. In addition, it brings interesting methodological alternatives for modeling country data such as using the GBD estimates at the country and subnational levels. Their results represent relevant subsidies for evidence-based policies to tackle NCDs.

An innovative approach was introduced in the paper “*Bi-objective goal programming for balancing costs vs. nutritional adequacy*” to deal with optimum lowest-cost and highest-quality diet planning (Koenen et al.). In their work, they present a method to find all trade-offs between any two linear objectives in a dietary linear programming context focusing on the trade-off between the costs of a diet and its nutritional adequacy. This method helps not only practitioners to understand better the possibilities for a diet that is as nutritious as possible within budgetary restrictions but the design of low-cost dietary interventions.

Finally, the paper “*Economic and health impacts of the Change4Life Food Scanner app: findings from a randomized pilot and feasibility study*” developed a pilot randomized controlled trial for evaluating clinical outcomes in children and economic effectiveness of the UK's Food4Life Food Scanner app, which is aimed at providing the nutritional content of packaged foods to families in order to encourage healthier diets among children (Mahdi et al.). Although modest mean differences between study arms were found over a short follow-up period, the study brought lessons on the feasibility of the strategy, yet it faces difficulties in obtaining data on app costs, and highlighted the importance of long-term economic modeling compared to short-term outcomes that may not be captured.

In summary, different methodologies for modeling the health and economic impact of risk factors and interventions can contribute to producing evidence for decision-makers in order to support more effective and cost-effective health policies in different countries that face common challenges in controlling and preventing diet-related diseases.

## Author contributions

EV: Conceptualization, Writing—original draft. IM: Conceptualization, Writing—original draft. EN: Conceptualization, Writing—original draft.
