# Corrigendum: Editorial: Economic and health impacts of dietary interventions

**DOI:** 10.3389/fnut.2023.1330432

**Published:** 2023-11-28

**Authors:** Eliseu Verly-Jr, Isis Eloah Machado, Eduardo Augusto Fernandes Nilson

**Affiliations:** ^1^Department of Epidemiology, Rio de Janeiro State University, Rio de Janeiro, Brazil; ^2^Medicine School, Federal University of Ouro Preto, Ouro Preto, Brazil; ^3^Núcleo de Pesquisas Epidemiológicas em Nutrição e Saúde, São Paulo, Brazil

**Keywords:** diet cost, Burden of Diseases, dietary interventions, cost-effectiveness, health impacts

In the published article, an author name Eliseu Verly-Jr. was incorrectly written as Eliseu Velry-Jr.

The authors apologize for this error and state that this does not change the scientific conclusions of the article in any way. The original article has been updated.

